# 'The Lost Senses'—Deafness

**Published:** 1846-01

**Authors:** 


### Art. XVI.

*The Lost Senses—Deafness.* By John Kitto, d.d. (*Knight's Weekly Volume for all Readers.*)—*London,* 1845. 12mo, pp. 206.

This is a unique book. The autobiography of a totally deaf man, of one whose "deafness could not be more intense had the organs conducive to the sense of hearing been altogether wanting." The writer is an author much esteemed, we believe, by biblical critics. His powers of minute observation, and of vivid description, and his vast amount of information are displayed in the notes to 'Knight's Pictorial Bible,' which are his writing, and this little work leaves a strong impression of his intellectual power. We learn from it that he has secured by his own exertions an acknowledged place among men of letters, and that moreover literature is the permanent employment to which he dedicates his life, and that it enables him, like any other successful profession, to support his family. But we pass, at once to his own story.

Dr. Kitto sprang from the very poor. His father was a jobbing mason, in precarious employment. He was slating a house. This son, a boy of 12, who, to the neglect of his school-education, lent "his small assistance," was carrying up a load of slates, and was stepping from the ladder to the roof when his foot slipped and he fell backward, thirty-five feet into the court below. He was stunned. For a moment he was conscious whilst his father, followed by a crowd, was carrying him home; but the succeeding fortnight was a blank in his life, and when he "awoke one morning to consciousness, it was as from a night of sleep." He attempted to spring up in bed, marvelling he had slept so late, and was astonished to find that he could not even move. He was slow in learning that his hearing was entirely gone. His utter prostration overcame any curiosity; and it was not until some time afterwards on inquiring for a book that had been promised to him, and was impatient at not comprehending the signs which were made to him, that one of his family wrote on a slate the awful words, "you are deaf." Awful, however, rather in retrospect, as he was a child, and knew not the future. "It was well I did not. It was left for time to show me the sad realities of the condition to which I was reduced." The recognized routine of remedies was tried. "They poured into my tortured ears various infusions, hot and cold; they bled me, they blistered me, leeched me, physicked me; and, at last, they put a watch between my teeth, and on finding that I was unable to distinguish the ticking, they gave it up as a bad case, and left me to my fate." Subsequently a seton in the neck, and electricity were tried, but "no good came of it." And with good sense he adds, "Indeed I have not sought any relief; and have discouraged the suggestions of friends who would have had me apply to Dr. This and Dr. That. The condition in which two thirds of life has been passed, has become a habit to me—a part of my physical nature. I have learned to acquiesce in it; and to mould my habits of life according to the conditions which it imposes; and have hence been unwilling to give footing for hopes, and expectations, which I feel in my heart can never be realized."

It was some time before he could leave his bed, and much longer before he could quit his chamber. Reading was his only resource. Before his accident he had been a lover of books; but this long spell at it went far to fix the habit of his future life, and the book he read ('Kirby's Wonderful Magazine,') was well calculated from the strange facts it recorded to draw his attention "to books as a source of interest and a means of information, and this was precisely the sort of feeling proper for drawing me into the habits which have enabled me, under all my privations, to be of some use in my day and generation."

At this period the art of reading was not diffused among the poor. "Many could read; but the acquirement was not in the same degree as now applied to practical purposes. It was regarded more in the light of an occult art—a particular and by no means necessary attainment, specially destined for and appropriate to religious uses, and Sunday occupations." The books at his command were but few and chiefly religious ones. The Bible, which he read quite through; Fox's Book of Martyrs, Josephus, Bunyan, Baxter, Sturm, and a few more. Good books all, "but the thing was you could see no other books than these." Periodical and daily literature, now so abundant had not then flowed over the land. His instinctive taste and an existence solitary and cut off from all social enjoyments by deafness, led him to read much, and his circumstances compelled him to read a few books. Thus by necessity he was obliged to comply with the good rule, to read much in a few good books. He was unconsciously drilling himself in the best habits of mind—application to a few subjects. Winchester or Eton might not have done more for him.

"The day came," he adds, "when I plunged into the sea of general literature, and being able to get nothing more to my mind, read poems, novels, histories, and magazines without end. Another day came, in which I was enabled to gratify a strange predilection for metaphysical books; and with all the novelists, poets, and historians within the reach of my arm, gave my days to Locke, Hartley, Tucker, Reid, Stewart, and Brown. I think little of these things now, and my taste for them has gone by; but although I now think that my time might have been more advantageously employed, my mind was doubtless thus carried through a very useful discipline, of which I have since reaped the benefit. But amid all this, the theological bias, given by my earlier reading and associations remained; and the time eventually came, when I was enabled to return to it, and indulge it with redoubled ardour; and after that, another time arrived, when I could turn to rich account whatever useful thing I had learned, and whatever talent I had cultivated, however remote such acquirement or cultivation might at first seem removed from any definite pursuits."

Having commenced with this sketch of his early life and mental training, he digests the remainder of his information under such heads as appear to him "calculated to develop the more striking facts and circumstances in the physical, social, and intellectual condition of one who has the chief entrance to his inner being closed."

The first chapter is on "Speech." It is the common opinion that the deaf and dumb are dumb because they are deaf. Our biographer believes from his own experience that there is a physical impediment to speech as well as to hearing. Before his fall his enunciation was remarkably clear and distinct, but after that he spoke with pain and difficulty, so as not to be easily understood, and he was told his "voice had become very similar to that of one born deaf and dumb, but who has been taught to speak."

"Although I have no recollection of physical pain in the act of speaking, I felt the strongest possible indisposition to use my vocal organs. I seemed to labour under a moral disability, which cannot be described by comparison with any disinclination which the reader can be supposed to have experienced. The disinclination which he feels to leave his warm bed in a frosty morning, is nothing to that which I experienced against any exercise of the organs of speech."

The force of this tendency to dumbness was so great that for years he habitually expressed himself in writing, and it was owing to the kindness of two passengers, his companions during a voyage, who,—seeing that dumbness was merely a bad habit, entered into a conspiracy with the captain to understand nothing except what was spoken, that made a wholesome compulsion,—he again expressed himself orally in the ordinary intercourse of life. At first he was understood by strangers with difficulty, but practice has greatly improved his speech. The following remarks were written by a friend on it:

"It is pitched in a far deeper bass tone than is natural to men who have their hearing. There is in it a certain contraction of the throat analogous to wheezing; and altogether it is eminently *guttural*. It may be suspected that this is attributable to the fact that his deafness came on in boyhood, before the voice had assumed its masculine depth. The transition having taken place without the guidance of the ear, was made at random, and without any pains bestowed upon it by those who could hear and correct it."

His tendency is to express words which he has acquired since his deafness as they are written. This renders his English often more intelligible to foreigners than to his countrymen. To his own sensations he seems to speak in a loud whisper, but from the effect on others he finds this is not the case, but that his voice is loud and strange. It may be heard as a sound to an unusual distance, although the articulations can only be made out by a person quite near. He thus graphically describes its effect:—

"If I happen to forget myself, and speak to a companion while others are walking just before us, it is equally annoying and amusing to see the sudden start and abrupt turn of the persons behind whom we walk, at a sound seemingly not of this earth, and so much beyond the range of all ordinary experience."

A friend has described his voice as if formed in the chest, and issuing through a tube without being much modulated by its passage through the mouth,—a definition which agrees well with his own sensations.

This unwillingness to speak is a common attendant on deafness, arising probably from many causes. Dr. Kitto is fully persuaded that in his case there was a physical inability to speak, and he is so accurate and truthful an observer that his impression on this head is good evidence of the fact. As the structure of his vocal organs was perfect before the accident, we have to look at the nerves alone for any physical causes of the inability. The injury to the brain at the origin of the auditory nerves, which produced the deafness, might have extended to the roots of the nerves which supply the muscles engaged in the production of the voice. And as the difficulty seems to be in modulating the voice, and not in producing the sound, the nerves supplying the tongue and lips might have been most jarred. "This physical difficulty in the formation of articulate sounds," of which Dr. Kitto is fully persuaded, however, is explicable on the greatly increased difficulties to the education of the voice which deafness produces. We learn to speak from imitating those we hear. Remove an English child to France, and in a very short time he speaks his own language with difficulty, and even forgets it. A child who suddenly becomes totally deaf is just in the condition to forget his own language without learning another, and if, as in Dr. Kitto's case, his parents' are poor and leave him much to himself and his books, he would in a short time have as much difficulty in speaking his own language as if he had been wholly among those who spoke another tongue. If in addition to this cause it is taken into consideration that he has lost by deafness his chief guide in the modulation of his sounds, and therefore the difficulty of speaking is very much increased, it is not surprising that his unwillingness to speak becomes a confirmed habit, and that in the course of time the difficulty in utterance becomes so great as to require great exertion and perseverance to overcome. Besides, the mutual cooperation of ear and voice in one act is at an end; this harmony is lost, and the pleasure that arises from it (the pleasure which each man derives from his own voice, musical to him, however discordant to his neighbour) is gone. That great spur to conversatiou, the love of approbation, can actuate him no longer from whom all social amusements are cut off, and who is compelled to find his own pleasures, not among his kind, but in the world of silent nature, of books, and of his own thoughts. It would be strange, therefore, if want of practice in speech, with absence of stimulus to its exertion, and of pleasure in its performance, did not very soon lead to an inability in the formation of sounds, as hard to be overcome as a bodily imperfection. Some letters and private memorandums of Beethoven (who, from possessing a most exquisite ear, the delicacy of which, he says, few could imagine, became deaf at thirty) reveal the mental sufferings and mortifications he underwent, which were set down to misanthropy and moroseness. He also avoided speaking as much as possible.

Swift, who became deaf, did not speak more than once during the two last years of his life.

It is a striking proof of Dr. Kitto's powers of observation that he forms a distinct impression in his own mind of the voices of those who speak in his presence.

"The impression thus conveyed is produced from a cursory, but probably very accurate observation of the person's general physical constitution, compared with the action of his mouth, and the play of his muscles in the act of speaking. I form a similar idea concerning the laugh of one person as distinguished from that of another; and when I have seen such a person laugh, the idea concerning his voice becomes in my mind a completed and unalterable fact. The impression thus realized would seem to be generally correct. I have sometimes tested it by describing to another the voices of persons with whom we were both acquainted, and I have not known an instance in which the impression described by me has not been declared to be remarkably accurate. This faculty must be based upon experience acquired during the days of my hearing, and cannot be realized by the born deaf, seeing that it is impossible for them to have any idea of sounds produced by the action of the vocal organs, and still less of the peculiarities by which one voice is distinguished from another."

Dr. Kitto alludes to the power which some deaf persons are said to possess of reading off the words of another from the motions of his lips. A case had been mentioned to him "by an intelligent stationer, of a lady who came to his shop and asked for certain articles in what seemed to him a foreign accent, and who so readily understood and replied to what he said, that he had not the slightest suspicion of her being deaf and dumb. This was, however, a lady of fortune, who had from childhood been the sole object of attention to an experienced governess, which may go far to account for her extraordinary power."

"I can myself," says Dr. Kitto, "make out some single words and short sentences in this way, when I know that what is said must be one of three or four things. But I am thoroughly persuaded that this mouth-reading must be wholly inadequate to the purposes of *real* conversation, involving intercourse of the intellect and imagination."

The next subject is "Percussion." The loudest thunder is perfectly inaudible to Dr. Kitto; beneath the full peal of a magnificent set of bells he is unconscious of any sound; he has been in a besieged city, but heard neither the cannon nor the shells. But there is in his whole frame a peculiar sensibility to the slightest percussion, producing a sense of vibration, pervading the whole frame "to the very bones and marrow."

"When a percussion of any kind takes place upon the very framework with which my standing-point is connected, the sensation is powerful, and the intensity is in the ratio of the closeness of the connexion. The drawing of furniture, as tables or sofas, over the floor above or below me, the shutting of doors, and the feet of children at play, distress me far more than the same causes would do if I were in actual possession of my hearing. By being to me unattended by any circumstances or preliminaries, they startle dreadfully; and by the vibration being diffused from the feet over the whole body, they shake the whole nervous system, in a way which even long use has not enabled me to bear. The moving of a table is to me more than to the reader would be the combined noise and vibration of a mail-coach drawn over a wooden floor; the feet of children like the tramp of horses upon the same floor; and the shutting of a door like a thunder-clap, shaking the very house."

His wife has observed that the lightest foot-fall upon the same floor is quite sufficient to attract his attention and even to rouse him from sleep. He is not conscious of knockings at the door however violent, while the shuttings of the door are painfully distinct.

"The reason of this is obvious; the valve of the door on which the percussion is made by knocking, is a detached frame of wood hung upon hinges, and the vibration is therefore comparatively isolated, and not propagated throughout the frame of the house, as is the case when, in shutting the door, the valve itself strikes upon the doorpost, which is identified with the framework of the building."

This sensation gives him no direction as to the place from which the sound proceeds; whether it is on the same floor, or above or below it. If a thimble, pencil, or even a more minute object falls from the table to the floor, he is often aware of it when others sitting at the same table have not been apprized of it by the ear. The greater number the points of contact with the floor the stronger the impressions, hence they are more distinct when he sits than when he stands.

"And when the chair itself in which I am seated has been subject to the percussion, the sensation is such as baffles description. For instance; a few days since, when I was seated with the back of my chair facing a chiffonnier, the door of this receptacle was opened by some one, and swung back so as to touch my chair. The touch could not but have been slight, but to me the concussion was dreadful, and almost made me scream with the surprise and pain—the sensation being very similar to that which a heavy person feels on touching the ground, when he has jumped from a higher place than he ought. Even this concussion, to me so violent and distressing, had not been noticed by any one in the room but myself."

Although of course insensible to the sound of music, he has derived agreeable sensations occasionally from the vibrations of stringed instruments, and especially from the higher notes of the piano.

"I found that the notes were the most distinct to me when the points of my finger-nails rested upon the cover, and still more when the cover over the wires was raised, and my fingers rested on the wood over which the wires are stretched, and to which they are attached."

He has not cultivated this means of imperfect gratification. When blowing in wind instruments he is not aware of any sound.

We have given Dr. Kitto's experience on percussions fully, and in his own lucid language, as it is of much interest as he describes a class of symptoms which are we suspect very common, with an accuracy and minuteness which is original. Since reading them we are reminded of similar complaints from these causes made by more than one deaf person, whose nervous system was delicately strung; often giving rise to difficulties in explaining their consciousness as to some trifling sounds, with their total incapacity in hearing louder ones. Another, and perhaps a larger class of invalids are very subject to the same kind of annoying impressions, those suffering from nervous complaints, women especially, whose originally delicate nervous organizations are rendered more susceptible by disease. Any jars from doors or footsteps are to such the source of positive suffering which they cannot control; but as, at the same time, their sense of hearing is often morbidly acute, the two classes of effects have not been hitherto accurately separated, all the annoyance perhaps being attributed to the sound when the jar of the easily-moved nervous system was the chief source of wretched feeling. The sufferers themselves are often well aware of the two causes.

We next come to his observations on *sight.* That the loss of one sense is compensated by the improvement in another, is not true physically in Dr. Kitto's case, unless his increased susceptibility to percussions may be so reckoned. His eyesight was not rendered more acute nor lasting. But mentally there is a compensation, and Dr. Kitto's close observation of his own mind, has enabled him to realize and to explain the effects upon it of the loss of one sense, and these materials towards the "philosophy of deafness" we gladly use. In the first place, it has developed "a sense of the beautiful in nature and art, and a love for it, a passionate love—which has been a source of his most deep and pleasurable emotions." This was owing to no æsthetic cultivation; no one suggested to him that such and such things were beautiful. His taste therefore "was more of the nature of instincts." It began to show itself soon after his accident "in a rapidly increasing admiration, and love of whatever gratified the eye, and a more intense abomination of whatever displeased it." The latter feelings were chiefly confined to "dead animals, especially as exhibited in shambles, and to persons deformed, or exhibiting in their countenances traits or expressions which I did not approve. This feeling became at length almost morbid; and I felt thoroughly miserable when in the same room with an ugly old woman, or with a man exhibiting distorted or imperfect features, labouring under any sinister or malignant expression in his countenance. I used to feel a strong inclination to fly at them, and to drive them from me." His range of pleasurable sensations were much greater. The moon was an object of his just idolatry.

"I have no recollection of earlier emotions connected with the beautiful, than those of which the moon was the object. How often, some two or three years after my affliction, did I not wander forth upon the hills, for no other purpose in the world, than to enjoy and feed upon the emotions connected with the sense of the beautiful in nature. It gladdened me, it filled my heart, I knew not why or how, to view the 'great and wide sea,' the wooded mountain, and even the silent tomb, under that pale radiance. This is one of the enjoyments of youth, which has not yet passed away. After this, I do not know that any class of objects in nature has acted so strongly upon my sense of the beautiful, or perhaps I should say of the sublime, as mountains. For to me high mountains were a feeling, from the time that I first gazed upon the glory of the Grenada mountains, as the sun cast his setting beams upon their tops, to that in which I caught the Titanic shadow of Etna in the horizon, or spent my days among the glories of the Caucasus, or wondered at the cloudy ring of Demavend, or mused day by day upon the dread magnificence of Ararat."

His enjoyment of trees was a rather later pleasure. There were but few in his native place by the sea-side, which he did not quit until twenty years of age. Travel perfected this enjoyment. The magnificent woods of England "called into full activity that perception of beauty in trees, which afterwards ministered greatly to my enjoyment as I travelled among the endless fir woods of northern Europe, and the magnificent plane trees of Media, and dwelt amidst the splendid palm groves of the Tigris." Since then I have seldom enjoyed serenity of mind in any house, from which a view of some tree or trees could not be commanded." His residence has been chosen with this view, and his study selected—

"Not for the room that might be in itself the most convenient, but the one from the window of which my view might, with the least effort, rest upon trees, whenever the eyes were raised from the book I read, or from the paper on which I wrote. In all cases, even the stillness of a tree has been pleasing to me; and the life of a tree, the waving of its body in the wind, or the vibration of its leaves and branchlets in the breeze, has been a positive enjoyment, a gentle excitement, under which I could have rested for hours. This strong feeling has enabled me to understand better than I otherwise might, the curious and often beautiful superstitions and idolatries which were associated with trees in the ancient times; and I have understood better than Aelian the class of associations which may have induced the Persian king to present the glorious plane near Sardis, with costly gifts, and to deck it with the ornaments of a bride."

That one thus thrown on his eye-sight for his pleasures should have early delighted in pictures, should have studied and got by heart each print in the scanty book-shop windows of his native place, and have subsequently found in the print-shops of London endless gratification, and that the National Gallery should have afforded him many happy hours, when in feasting his eye and imagination he purified his tastes, and strengthened his perceptions, is to be expected. That he is an accurate physiognomist at first sight is probable enough also, even although he is unskilled in the science of Lavater or Spurzheim, and the busy crowded thoroughfares and large assemblies furnish him with living pictures which he delights in scrutinizing. Amongst the "masses of ill-compacted matter, which has been cut up to form the aggregate of the insipid and characterless faces which crowd our streets," some one face really beautiful or ugly, really striking or repulsive, may be found to recompense the toil. Another compensation in his loss is the distinctness and permanence with which images received through the eye are impressed on his mind, and which as he suspects may be greatly owing to the eye being the sole channel through which images of objects have access to his brain.

"It thus happens that my mind retains a most distinct and minute impression of every circumstance in which, at the time of occurrence, I felt the slightest degree of interest; of every person whom I have at any time during the last twenty-eight years regarded with more than casual observation; and of every scene upon which, during frequent and long-continued change of place, I bestowed more than cursory notice."

By "cursory notice," he means seeing things without looking at them. This faculty is of much use and solace, for he calls up scenes with the *whole* of their circumstances.

"If I wish to recollect a person, along with him comes all the scenery amidst which I beheld him, and all the persons who were at the same time associated with him; and so, in like manner, if I wish to realize a scene, to conjure up the view of a place, it comes before me with the very persons I saw in it, and in all the identifying circumstances of form, and feature, and relative position. In actual travel I was loth to trust to a faculty which had not been sufficiently tried. I therefore diligently wrote up my travelling journals day by day. But although I had much occasion for the literary use of the facts and observations thus obtained and preserved, I have had scarcely any need to *refer* to these journals, seeing that whatever I wished to recollect became at once present to my mind in all its accessories and circumstances."

This distinctness cannot be referred to journalizing, as the recollections are equally clear as to one part, of 500 miles, where no journal was kept, and for home scenes.

That the power of recollection and of bringing up scenes so vividly and minutely, is a very uncommon one as to degree, self-observation, as well as observations of the impressions retained by others, daily and hourly testifies. Dr. Kitto's descriptions of the scenes, manners, and people of the east struck us before reading this autobiography, from their vividness, minuteness, freshness, and fulness. The impression was, " this is not a compilation, the writer has been at the spot with a keen practised eye." Such a power to such a degree, enabling him to make his mind " a temple for all lovely forms," seems almost a sufficient compensation for his loss. But the acuteness of sensibility which is the attendant of such gifts, renders their possessor more alive to his own deficiencies. Dr. Kitto does not shirk this painful subject, but next passes to his " disqualifications." This part, admirable though it be and worth reading and reflecting upon by all who are struggling onwards, is not so immediately related to the subjects we discuss as to admit our dwelling fully upon it. Even if it were, we could not analyse or compress what is so compact, and full of thoughts in consequence. The book is but a shilling. Buy it. Twelve pence is not ill spent, in seeing how a vigorous mind struggles through difficulties, all but insuperable; that makes its own way, not only through poverty, and want of sympathy, and absence of appreciation, and actual discouragement from well-intentioned friends, but also in spite of a physical defect which increased these hinderances to success a hundredfold, until it attained its right position among its fellows, and thereby content, bearing witness that " there is no one so low but that he may rise; no condition so cast down as to be really hopeless; and no privation which need of itself shut out any man from the paths of honorable exertion, or from the hope of usefulness in life."

To trace his progress, to see his pure love of knowledge for its own sake, driving him on and keeping him up to constant exertion with a tension which needed none of the stimulus of prizes, or even of fellow-feeling, to see him humbly submitting to employments against his tastes, and in which he could not place his heart, but which never led him astray from his one object, until at last, casting all other cares aside, and determining at all risks to act upon his " soul-felt conclusions, and to stand by the truth or fall by the error of his ineradicable convictions," and to learn that he has succeeded, must do all readers good. For his success has been won by unremitting toil, and his own strong instincts led him not to the mere gratification of his tastes, but into a path of earnest and useful labour, which he felt he had been prepared for walking in.

Dr. Kitto communicates with his own household, and with all those with whom he is on terms of personal intercourse, by finger-talking. Those interested either for themselves or for others in this art, would do well to consult this book, as his hints as to its practice must be useful. Of signs he has personally a great aversion, from their attracting the attention and curiosity of others, but he is fully convinced, that signs are the natural language of the deaf and dumb, and that time is thrown away in teaching them to speak. A difference of opinion exists on this point. The Abbé Sicard taught few of his pupils to speak. They have a great aversion to it, whilst they communicate with each other by signs, with an hilarity and " abandon" very exhilarating to witness. As Dr. Kitto observes, they must ever be cut off from *oral* intercourse with society, and that "education therefore is the best which creates the highest appetite for books, and which, by conferring a complete mastery of written language, not only gives them as much intercourse with the minds of others as they are capable of enjoying, but opens up to them the wide world of facts and ideas which books contain."

His chapter on "society" is excellent. Full of nice discernment, genuine modesty, an acute appreciation of what is due to others, with indications of shyness and reserve, which his early life, studious habits, and his malady must have encouraged. With the full sense of the terrible privation he endures, and with every evidence from his entering into the subject minutely, that he has sharply felt its misery, yet the humour which everywhere gleams and enlivens his narrative, shows that he has neither become misanthropical, cynical, nor even stoical. But with manly good sense has learned, that the true art of enduring, is in submitting unconditionally to the will of Providence. He very judiciously says,

"It is surely a social duty in the deaf to avoid company, in the assurance that by going into it, or gathering it around him, he is only a stumbling-block to the pleasures of others, and is only laying up for himself a store of mortification and regret for those terrible disqualifications, which, in the solitude of his chamber, or in the presence of his trained domestic circle, he may half forget."

Dr. Kitto after concluding his own history, comments on that of Massieu, (the celebrated deaf-and-dumb pupil of the Abbé Sicard,) whose beautiful definition of gratitude, "the memory of the heart," has immortalized him. The following questions were put to him:

"Did you know what it was to hear?

"Yes.

"How had you learned that?

"A hearing female relative told me that she saw with her ears a person whom she could not see with her eyes; who was coming to my father. The hearing *see with their ears* during the night a person who is walking."

This answer is very analogous to that of the blind man, who explained his notion of scarlet, by comparing it to the sound of a trumpet. The blind man comparing a sight to a sound, whilst the deaf man compared sounds to sights. As Dr. Kitto says, the blind believe that sight is a kind of ocular hearing; and the deaf, that hearing is a kind of auricular sight. Dr. Kitto thinks, that the deaf-and-dumb believe there is a certain emission from the lips in motion, visible to others and not to themselves.

"Meanwhile it is certain that no *proper* idea of a sound can be entertained by those who never heard it, whatever be the degree of their education. The nearest approach to such an idea which they can possibly reach, is through the class of hitherto undescribed sensations which I have endeavoured to explain in the chapter on percussions."

This strikes us as a very acute and true remark, and a new one. Physiology confirms it, as both sensations depend on vibrations.

In bringing to an end our analysis of this 'lay' book, we trust that the pleasure we have taken in analysing it, will be conveyed in some measure to our readers, and will induce them to peruse the little volume itself. In a purely scientific point of view, it contains original matter, the result of the careful observation and the personal experience of many years, by one who can observe, and vividly convey his impressions. The remarks on percussions, as well as on the effect of the loss of hearing on the development of those mental tastes which depend on the eye, and the high spiritual compensation thus afforded, rather than a physical one in the mere strengthening of the power of other senses, are worthy the attention both of the physiologist and metaphysician, and are proofs of the principle which in this review has been always strenuously insisted on, that the two studies should be united in order to throw any new light on that deep branch of inquiry. But besides its scientific value, another lesson may be incidentally learned from it;—the history of a manly, honest, earnest mind, pursuing knowledge from the pure love of it, without any ulterior view, and under difficulties seemingly overwhelming, and this perseverance ending (as perseverance does) in success, although that success could not have been foreseen, is good as an example in a profession like our own, where the scientific objects are so high, the difficulties to be overcome so numerous, and the attractions to take a shorter and easier road than persevering labour points out, are so numerous and seducing. The strong will, the self-dependence gradually growing out of and based firmly (as the event proved) on an accurate self-knowledge, and not on an inflated estimate of his own capacities; the freedom from vanity which this shows, and with it genuine humility, and self-distrust under new circumstances which may happen, and of which he has had no experience, and the feeling of satisfaction and content, his honest pride in having used his success to his own exertions, and yet his submission to, and acknowledgment of a guiding Providence, are traits of a character which cannot be studied without the conviction that it is a high one, under a wish (which may not be barren) that such was the reader's own.

---

